# Evaluation of a modified clinical prediction rule for use with spinal manipulative therapy in patients with chronic low back pain: a randomized clinical trial

**DOI:** 10.1186/s12998-014-0041-8

**Published:** 2014-11-18

**Authors:** Paul E Dougherty, Jurgis Karuza, Dorian Savino, Paul Katz

**Affiliations:** Canandaigua Veterans Affairs Medical Center, Canandaigua, NY USA; New York Chiropractic College, Seneca Falls, NY USA; University of Rochester School of Medicine and Dentistry, Rochester, NY USA; University of Rochester, Rochester, NY USA; State University of New York College at Buffalo, Buffalo, NY USA; University of Toronto, Toronto, ON Canada; Medical Affairs, Baycrest Geriatric Centre, Toronto, Canada

**Keywords:** Clinical prediction rule, Chronic lower back pain, Spinal manipulative therapy, Active exercise therapy, Randomized controlled trial

## Abstract

**Background:**

Spinal Manipulative Therapy (SMT) and Active Exercise Therapy (AET) have both demonstrated efficacy in the treatment of Chronic Lower Back Pain (CLBP). A Clinical Prediction Rule (CPR) for responsiveness to SMT has been validated in a heterogeneous lower back pain population; however there is a need to evaluate this CPR specifically for patients with CLBP, which is a significant source of disability.

**Methods:**

We conducted a randomized controlled trial (RCT) in Veteran Affairs and civilian outpatient clinics evaluating a modification of the original CPR (mCPR) in CLBP, eliminating acute low back pain and altering the specific types of SMT to improve generalizability. We enrolled and followed 181 patients with CLBP from 2007 to 2010. Patients were randomized by status on the mCPR to undergo either SMT or AET twice a week for four weeks. Providers and statisticians were blinded as to mCPR status. We collected outcome measures at 5, 12 and 24-weeks post baseline. We tested our study hypotheses by a general linear model repeated measures procedure following a univariate analysis of covariance approach. Outcome measures included, Visual Analogue Scale, Bodily pain subscale of SF-36 and the Oswestry Disability Index, Patient Satisfaction and Patient Expectation.

**Results:**

Of the 89 AET patients, 69 (78%) completed the study and of the 92 SMT patients, 76 (83%) completed the study. As hypothesized, we found main effects of time where the SMT and AET groups showed significant improvements in pain and disability from baseline. There were no differences in treatment outcomes between groups in response to the treatment, given the lack of significant treatment x time interactions. The mCPR x treatment x time interactions were not significant. The differences in outcomes between treatment groups were the same for positive and negative on the mCPR groups, thus our second hypothesis was not supported.

**Conclusions:**

We found no evidence that a modification of the original CPR can be used to discriminate CLBP patients that would benefit more from SMT. Further studies are needed to further clarify the patient characteristics that moderate treatment responsiveness to specific interventions for CLBP.

**Trial registration:**

ISRCTN30511490

## Background

Chronic lower back pain (CLBP) is a significant public health problem in both Veterans and the general population [1,2]. Chronic Lower Back Pain is not only a problem in the US, the recent global burden of disease reports that it is one of the most common causes of years lived with disability [2]. Chronic lower back pain is secondary only to respiratory conditions in reasons for visiting primary care [3]. Despite over 200 treatments for CLBP, the costs of treating CLBP have risen 65% in the last 10 years with no appreciable improvement in patient outcomes [4,5].

One contributory factor is inappropriate management due to poor understanding of prognostic factors [6,7]. This is particularly relevant for primary care providers who must make decisions on management strategies for this very common problem [8]. Spinal Manipulative Therapy (SMT) and Active Exercise Therapy (AET) are two commonly utilized, evidence based, interventions for CLBP [9] however neither has shown superiority [10]. It has been hypothesized that identification of specific characteristics predicting clinical responsiveness to these interventions would improve the outcomes through appropriate management [11].

The desire to identify these specific patient characteristics has led to the development of clinical prediction rules (CPR). A CPR is a clinical tool that quantifies individual contributions that various components of the history as well as the physical examination results make towards the diagnosis, prognosis, or likely response to treatment in an individual patient [12]. The CPR for SMT (CPR SMT) was first reported in 2002 [12] and then a validation study was published in 2004 [13]. This CPR predicted responsiveness in patients with lower back pain (LBP) to SMT [13]. Although this is the most studied of the CPRs, it still has not achieved the level of validation to be recommended for general clinical practice [14]. Furthermore, the previous validation studies of CPR SMT included acute, sub-acute and chronic conditions, and so their generalizability specifically to CLBP is unclear. A recent systematic review stated that there “is a lack of good quality RCTs validating the effects of a clinical prediction rule for low back pain” [15].

The current study evaluates the generalizability of the CPR for SMT to a CLBP population. The current study modified the original CPR for SMT for use with a CLBP population therefore creating a modified CPR for SMT (mCPR) that applies four of the five originally proposed components of the CPR for SMT.

### Study hypotheses

Based on previous data, our first hypothesis is that patients in both SMT and AET groups would demonstrate statistically and clinically significant improvements in disability and pain from baseline [9,10]. Our second hypothesis predicts that the mCPR moderates the comparative effectiveness of treatment in the SMT group but not the AET treatment group. Based on the data from Childs et al. [13], we hypothesized that the mCPR modifies the comparative effectiveness of (i.e., the differences between) the two treatment groups. We expect the comparative effectiveness between the two treatment arms for the positive on the mCPR group would be different from the comparative effectiveness between the two treatment arms for the negative on the mCPR group.

## Methods

### Trial design

The study was a prospective RCT using a stratified permutated block design conducted between 2007 and 2010. Chronic lower back pain patients were recruited and evaluated for their status on a mCPR for responsiveness to SMT. Patients were then randomized to receive either SMT or AET twice a week for four weeks. The protocol received Institutional Review Board approval through the Syracuse/Canandaigua Veterans’ Affairs (VA) Medical Centers (MIRB#00367) and through the New York Chiropractic College (NYCC) (IRB#07-01). Trial Registration: ISRCTN30511490- http://www.controlled-trials.com/ISRCTN30511490/.

### Participants

Our patients were 181 adults who met the following inclusions criteria. Inclusion: LBP for ≥12 weeks prior to enrollment, pain upon deep palpation of the lumbar erector spinae, LBP from L1 to sacroiliac joint inclusive, live within 50 miles of Rochester, NY, have a baseline >30 mm on the Visual Analogue Scale (VAS) [16] and >20% on the Oswestry Disability Index (ODI) [17]. Patients had to be willing to undergo no new or different treatment during the study intervention and follow up period, although they were allowed to continue any medications.

Exclusion: Radiographic or clinical evidence of cauda equina syndrome, spinal neoplasia or metastatic disease, destructive joint pathology such as rheumatoid arthritis, bowel/bladder dysfunction associated with the LBP, peripheral neuropathy or progressive lumbosacral radiculopathy, progressive myelopathy or neurogenic claudication and spinal surgery within the past six months. Patients were excluded if they had undergone a course of SMT or supervised AET within the six months prior to enrollment into the study and if they could not perform an exercise program based on a New York Heart Association Classification of grade III or IV [18].

To attempt to reduce any variability of the assessment of the mCPR physical examination elements, all study patients were screened by the same clinician, the VA patients were screened in a VA setting and the non-VA patients were screened at a local hospital clinic. Based on the response to the Fear Avoidance Belief Questionnaire (FABQ), subjective symptoms and the physical exam findings, patients were categorized in terms of whether they were positive or negative on the modified clinical prediction rule [13]. Patients within each group were then randomized into either the SMT or AET treatments.

### Study settings

This multisite RCT was conducted in Rochester, NY at the VA Outpatient chiropractic and physical therapy clinics and two civilian outpatient chiropractic clinics and two civilian outpatient physical therapy clinics. The SMT interventions were carried out by licensed chiropractors (DC) and the AET interventions were carried out by licensed physical therapists (PT) at both the VA and in the private locations. Prior to initiation of the study the providers (DC and PT) met to discuss evaluation and treatment parameters and a video was made as a resource for all providers to refer to if they had questions.

### Interventions

Spinal Manipulative Therapy, as defined in this study, included high velocity low amplitude spinal manipulation and/or flexion distraction therapy or mobilization, and advise on heat/ice all of which are commonly performed by manual therapists [19-21]. The practitioner was allowed to give the patient one of two stretches to do at home, either “cat/camel stretch” or “knee to chest stretch.” While this definition differed from the original CPR validation study, it was felt that this allowed for greater generalizability of the SMT arm of the study to those who perform manual therapy.

Active Exercise Therapy included directional preference exercises, lumbar stabilization, general flexibility, and specific training exercises [22-24]. The therapists were given freedom to choose the active care exercise that they felt was best suited to the patient’s needs, but could only utilize those exercises that were included in the protocol and no specific limit was given on the number of exercises that could be prescribed. No passive stretching or modalities such as electric stimulation or ultrasound were allowed. Both treatment were performed twice a week for four weeks [25].

### Outcomes

The study evaluated improvement in pain using the VAS [16] and the SF-36 pain subscale [26] and disability using the ODI [17] in CLBP patients. The treatment outcomes are described in Table 1. All outcome measures have previously been validated in a CLBP population. Outcome measures were collected at baseline, 5, 12 and 24-weeks post baseline. A face valid open-ended Patient Expectation scale was administered prior to and after randomization to detect potential patient bias associated with the assigned treatment intervention. This scale asked patients to “Circle below on the scale from 0 to 10 how confident you are that the treatment you will be receiving will be successful at reducing your low-back pain.” Responses were measured on an 11 point scale anchored by “Not Confident” and “Confident”. As an adjunct to the outcomes, we examined the patients’ satisfaction at the end of the treatment at the last data collection point, the 24-week follow up visit, by administering a Patient Satisfaction survey [27]. We computed a mean patient treatment satisfaction score for each subject by averaging the ten patient satisfaction questions. Four of the items were reversed scored and were recoded so that the higher the score indicated the more satisfied the subject. The scale was reliable, (Cronbach’s alpha = .85). We performed a 2 × 2 analysis of variance on the treatment satisfaction score with the mCPR and treatment arm as the two between subject factors.

**Table 1:** **Summary of outcome measures**

**Variable**	**Definition**
VAS: Visual Analogue Scale	A patient completed analogue measure that evaluates pain intensity on a 100 mm long horizontal line.
ODI Oswestry Disability Index	The back pain specific, self-rating scale to measure the degree of functional impairment that a subject is experiencing in a number of activities of daily living.
Patient Satisfaction	A self-developed questionnaire based on Cherkin’s satisfaction questionnaire.
Patient Expectation	The recovery expectations measured using a time-based, specific single-item tool produced a strong prediction of outcome.
SF-36: Short Form-36 item health survey	A set of generic, coherent, and easily administered physical and mental quality-of-life measures.

### Randomization

Randomization to treatments was through a random number producing algorithm. The same screening clinician performed the history and physical exam for all study participants. The screening clinician was blinded to the results of the FABQ, and thus did not assign the status on the rule. The study coordinator administered the baseline questionnaires including: VAS, ODI, SF-36, FABQ and the Patient Expectation scale. The de-identified results of the FABQ and the subjective and objective components of the mCPR were faxed to the evaluator who combined the data and determined the status on the mCPR. Once the status was determined the evaluator utilized the random number algorithm to assign the intervention. The assignment of treatment intervention was then faxed to the study coordinator who scheduled the first visit with the appropriate provider. The screening clinician, the statistician and the treating clinician were all blinded to the status on the mCPR. The patient data on the mCPR was revealed upon completion of the study. At the first treatment visit, the patient was given the second Patient Expectation scale. Patients were recruited through radio ads, posters, and physician recruitment.

### Modified clinical prediction rule

The original study validating the CPR for SMT included acute, sub-acute and chronic lower back pain patients; however the median duration of pain for participants was 27 days [13]. Therefore, most of the patients in the original validation were classified as having acute and sub-acute lower back pain. Given that much of the morbidity associated with back pain is in chronic lower back pain patients, we felt that it was necessary to assess a modification of the rule in an exclusively CLBP population. The original mCPR criteria included: pain <16 days, pain proximal to the knee, internal hip rotation of greater than 35 degrees, hypomobility of one or greater lumbar segments and FABQ work subscale score of less than 19 [12]. The original rule was modified to exclude the criteria concerning pain <16 days, this allowed for only CLBP patients to be included. In order for a patient to be considered positive on the mCPR, they had to have at least three of the four criteria positive from the original rule.

### Power analysis

Paralleling Child et al’s study [13], we based sample size calculation on our primary outcome disability measure, the ODI. Based on the work of Ostelo et al. [28] a minimally clinically significant outcome for the ODI is 10 points. Our power analysis was based on detecting a difference that size or larger when comparing the effectiveness between the two treatment arms in both the positive on the mCPR group and negative on the mCPR group at each time of measurement which would be necessary in the test of our second hypothesis. We required 112 patients in a balanced design, or 28 patients in each group assuming an α level of 0.05, two tailed test, and a power (1-beta) of .80. Our power analysis for our secondary outcomes, the VAS, and SF-36 pain sub score, required the same or fewer patients to detect the same clinically meaningful differences. Since we treated them as secondary outcomes, we did not perform an alpha correction for the power analysis. Because of difficulties in recruiting patients who were negative on the mCPR and given our randomization strategy we were forced to recruit additional patients who were positive on the mCPR until we reached the minimum number of patients who were negative on the mCPR. Rather than exclude them from the analyses, and thus possibly create a bias, we kept the additional patients who were positive mCRP, which resulted in an excess of patients in the positive mCPR cells.

### Statistical analyses

We tested our study hypotheses a repeated measures analysis of variance strategy using Statistical Package for the Social Sciences (SPSS) version 21. The patients’ status on the mCPR (negative, positive) and the treatment group the patients were randomized to (SMT, AET) defined the between subject factors. The time of measurement (baseline, 5, 12 and 24-weeks post baseline) defined the within subject repeated measures factor. To control for age and length of pain significant differences and the fact that this was a multi-site study, we included them as covariates in the analyses.

We articulated our hypothesis in terms of main effects and interactions, as defined by our study design, and we employed traditional analyses of covariance tests of significance to test our derived hypothesis. We tested our hypothesis 1, which predicts significant clinical improvement in patients’ outcomes in both SMT and AET, by testing the main effect of time. We would expect a significant main effect of time, which would reflect a significant improvement in outcomes from baseline in both treatment groups. We also tested for mean differences between treatment groups in response to treatment over time, which is tested by the treatment × time interaction, to see whether the pattern of improvement was similar in the two treatment groups.

We tested our hypothesis 2, which predicts that the mCPR modifies the comparative effectiveness of (i.e., the differences between) the two treatment groups, i.e., the comparative effectiveness between the treatment arms for the positive on the mCPR group is different from the comparative effectiveness between the treatment arms for the negative on the mCPR group by examining the mCPR × treatment × time of measurement interaction.

We report the effect sizes using partial eta^2^. Partial eta^2^ is an effect size measurement for analysis of variance with more than one independent variable and conceptually is the proportion of variance in the dependent variable explained by an effect while controlling for other effects.

We used an intention to treat approach that included all enrolled patients who met inclusion criteria regardless of whether they completed the study. Ten patients dropped out. There was no significant difference in the dropout rate across the study groups. We used multiple imputation to handle missing data. We conducted the multiple imputation using SPSS missing values module (version 21) with five imputation runs using an iterative Markov chain Monte Carlo (MCMC) method with a linear regression model and assuming data missing at random.

We included the outcome measures at baseline and followups, subject expectations for treatment effectiveness, and the subject characteristics, with categorical variables dummy coded, as the variables in the multiple imputation.

## Results

**Figure 1:**
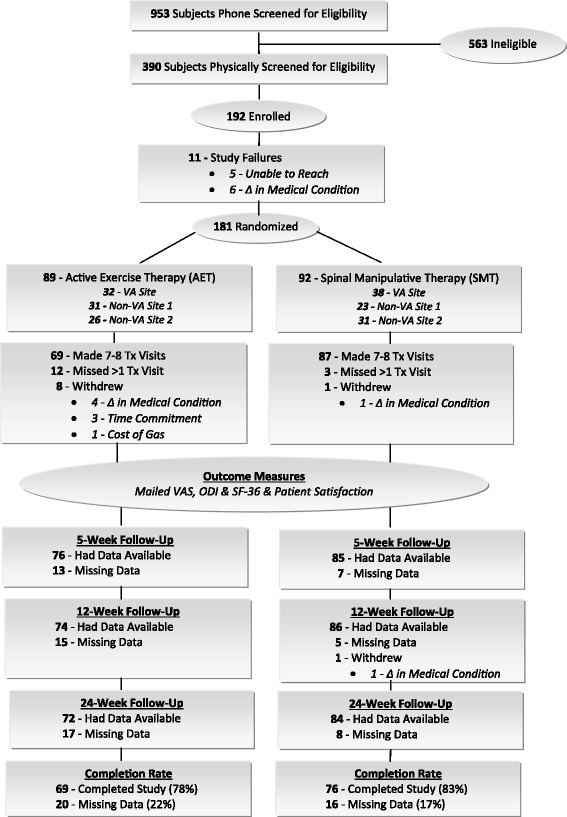
**Study flow sheet.**

A total 953 patients were phone screened of which 390 patients were physically screened. A total of 181 CLBP patients were enrolled; 89 were randomized into AET of these 69 (78%) completed the study and 92 to SMT of these 76 (83%) completed the study. (See Figure 1 for details).

### Patient characteristics

As seen in Table 2, the sample was predominately white, male, overweight, and with a mean age ranging from 53 to 61 years. Most attended college or graduated from college. While patients who were recruited from and treated in VA clinics accounted for slightly less than half the patients, the percentage of VA patients across groups was not significantly different. Slightly more than one third of the patients self-reported arthritis on their clinical history, and about one third of the patients self-reported a depression diagnosis. We did not find any significant differences between patients who were positive on the mCPR and patients negative on the mCPR with the exception of age and self-report of pain duration. We included these patient characteristics as covariates in the analysis.

**Table 2:** **Patient demographics**

**Variable**	**Study group**			
	**SMT**	**SMT**	**AET**	**AET**
	**Negative mCPR**	**Positive mCPR**	**Negative mCPR**	**Positive mCPR**
	**N = 32 (St. Dev.)**	**N = 60 (St. Dev.)**	**N = 28 (St. Dev.)**	**N = 61 (St. Dev.)**
Mean Age^a^	61.16 (16.24)	54.12 (16.04)	60.00 (15.00)	53.43 (18.18)
Mean Height (inches)	67.62 (4.14)	67.55 (4.08)	67.29 (3.36)	68.23 (5.82)
Mean Weight (pounds)	206.09 (39.36)	206.22 (49.34)	201.75 (34.07)	192.25 (44.31)
Mean BMI	31.84 (6.75)	31.71 (6.87)	31.34 (4.95)	29.34 (7.36)
Mean Pain Duration (mos)^b^	261.31 (237.90)	140.78 (196.29)	186.62 (188.63)	138.37 (152.13)
Arthritis	56%	40%	39%	38%
Osteoporosis	0%	2%	0%	0%
Depression	37%	45%	25%	28%
Female	25%	38%	24%	36%
White	91%	87%	82%	84%
African American	6%	8%	14%	11%
Hispanic	0%	5%	0%	5%
VA Patients	47%	37%	59%	30%
Education				
Some High School or Less	0%	5%	11%	8%
High School Graduate	41%	20%	18%	21%
Some College	31%	32%	43%	27%
College Graduate	8%	21%	26%	44%
Previous Treatment History				
Allopathic Medicine	87%	75%	93%	77%
Physical Therapy	41%	40%	52%	34%
Chiropractic	62%	55%	67%	52%
Surgery	16%	10%	26%	8%
Injection	12%	13%	26%	19%

^a^difference between positive and negative mCPR groups, p < .01.

^b^difference between positive and negative mCPR groups, p < .01.

Also seen in Table 2, nearly all patients previously sought allopathic medicine treatment for the CLBP and a majority had previously sought chiropractic treatment in all groups.

We next examined patient expectations of the treatment’s effectiveness prior to and after randomization to assure that patients who may have had preconceived biases toward one treatment or another would be detected, as this would have the potential to affect treatment responsiveness [29]. We performed a 4 × 2 analyses of variance with the study group as the between subject factor, and the time of the expectation rating (prior to randomization and post randomization before the start of treatment) as the within subject factor. We present the group × time interaction in Table 3. Patients became slightly more positive in their expectations for the treatment’s effectiveness after randomization to their treatment arm compared to their expectations prior to randomization (main effect of time, p = .02). This increase was similar across the four study groups, as indicated by the absence of a significant study group × time interaction (p = .43). This finding indicates that patients did not seem to lower their expectations once they knew which treatment they would be receiving.

**Table 3:** **Patient expectation**

	**Patient expectation**	
	**Mean** ^**a**^	**(±95%** **CI)**
**Negative on mCPR SMT (n = 32)**		
Pre randomization	5.78	(5.09, 6.47)
Post randomization	6.03	(5.27, 6.79)
**Negative on mCPR AET (n = 28)**		
Pre randomization	6.14	(5.41, 6.88)
Post randomization	6.79	(5.97, 7.60)
**Positive on mCPR SMT (n = 60)**		
Pre randomization	6.62	(6.11, 7.12)
Post randomization	7.07	(6.51, 7.62)
**Positive on mCPR AET (n = 61)**		
Pre randomization	6.46	(5.95, 6.96)
Post randomization	6.47	(5.91, 7.04)

^a^patient expectation for treatment score. The higher the number the more confident the patient was with the treatment. Responses measured on a 11 point scale anchored by 0, not at all confident and 10, very confident.

Given that this was a multisite study, before combining the data, we tested for the presence of site differences. We ran preliminary analyses that included site of treatment as a between subject factor. We found no significant site of treatment main effects or interactive effects of site of treatment with treatment group, mCPR, or time of measurement on the outcome measures.

With the absence of site of treatment effects, we pooled data across treatment sites. Even so, we included site as a covariate in our analysis.

We performed separate analyses on our three outcome measures, i.e., VAS, ODI, and the SF-36 pain subscales. We present the means and standard deviations for the outcome measures at each point in time in Table 4.

**Table 4:** **Intention to treat analysis**

**mCPR status treatment group time of measurement**	**Outcome measures**		
	**VAS** ^**a**^	**ODI** ^**b**^	**SF-36 Pain** ^**c**^
	**(St. Dev.)**	**(St. Dev.)**	**(St. Dev.)**
**Negative on mCPR**			
**SMT (n = 32)**			
Baseline	58.44 (15.46)	35.13 (8.55)	5.56 (1.20)
5-weeks	37.51 (28.89)	29.01 (14.66)	6.98 (1.66)
12-weeks	43.29 (24.62)	32.14 (15.77)	6.41 (1.92)
24-weeks	47.61 (25.66)	30.17 (15.69)	6.60 (2.06)
**AET (n = 28)**			
Baseline	65.36 (16.78)	37.04 (12.57)	5.35 (1.21)
5-weeks	36.50 (33.77)	30.15 (17.71)	6.56 (2.33)
12-weeks	42.00 (33.57)	32.91 (20.82)	6.54 (2.72)
24-weeks	52.54 (27.24)	32.71 (18.64)	6.44 (2.47)
**Positive on mCPR**			
**SMT (n = 60)**			
Baseline	61.25 (13.74)	33.62 (9.60)	5.78 (1.22)
5-weeks	34.86 (31.18)	26.71 (15.06)	7.10 (2.22)
12-weeks	40.43 (27.53)	29.64 (18.30)	6.57 (2.55)
24-weeks	38.47 (26.99)	23.16 (15.74)	7.51 (2.71)
**AET (n = 61)**			
Baseline	55.38 (16.64)	31.44 (10.05)	6.00 (1.52)
5-weeks	26.60 (33.48)	23.73 (17.14)	7.49 (2.57)
12-weeks	36.30 (29.64)	25.94 (19.90)	7.05 (2.98)
24-weeks	42.11 (31.77)	23.52 (18.98)	7.75 (3.08)

^a^the higher the number the higher the reported pain on a 100 point scale.

^b^the higher the number the more disability reported due to pain on a 100 point scale.

^c^the higher the number the *less* self-reported pain on the computed SF-36 pain subscale.

### Test of hypothesis 1

**Figure 2:**
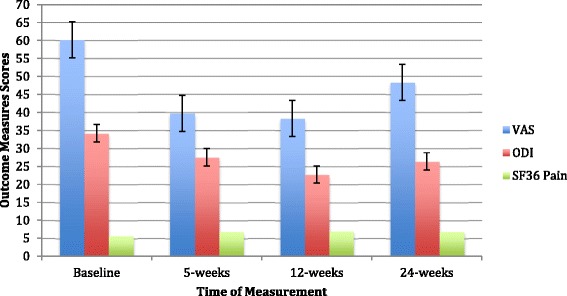
**Adjusted Outcome Measures.** VAS, ODI, and SF-36 Pain Subscale scores, adjusted for age, duration of pain, and treatment site, and 95% confidence intervals at baseline, post treatment 5, 12 and 24-weeks followup. Linear decrease significant for VAS (p = .03), ODI (p = .001) and SF-36 Pain Subscale (p = .002). The larger the score the more pain as measured by the VAS and disability as measured by the ODI. The larger the score the *less* pain as measured by the SF-36 Pain Subscale.

We found a significant time of measurement effect for VAS (p = .05, partial eta^2^ = .02), ODI (p = .001, partial eta^2^ = .04), and the SF-36 pain subscale (p = .003, partial eta^2^ = .03). We found no significant treatment arm × time interactions (p > .50). The SMT and AET groups both exhibited similar improvements in pain and disability outcomes after treatment. To help interpret the significant main effect of time, we then tested the within-patients linear, quadratic, and cubic contrasts with analysis of variance for each of the three outcome measures. We only found significant linear contrasts for the VAS (p = .03), SF-36 pain subscale (p = .002) and ODI (p = .001) with pain and disability dropping from the baseline to the post treatment follow ups (see Figure 2). The pattern of results support hypothesis 1.

### Test of hypothesis 2

We did not find significant mCPR × treatment × time of measurement interactions on the three outcome measures, VAS (p = .70), ODI (p = .76), and SF-36 pain subscale (p = .93). This is contrary to the expected pattern of results and so does not support hypothesis 2. We found no differences in the changes in treatment outcomes between SMT and AET in the positive on the mCPR group and no differences in the changes in treatment outcomes between SMT and AET in the negative on the mCPR group. We present the adjusted cell means and confidence intervals within the negative on the mCPR and positive on the mCPR groups for each of the follow up time points in Table 5.

**Table 5:** **Adjusted difference scores between SMT and AET groups**

**Time of measurement**	**Outcome measures**		
	**VAS** ^**a**^ **(±** **95%** **CI)**	**ODI** ^**b**^ **(±** **95%** **CI)**	**SF-36 pain** ^**c**^ **(±** **95%** **CI)**
**5-week Follow Up**			
Negative on mCPR between	7.16 (−20.32, 34.62)	0.75 (−8.84, 10.23)	0.21 (−1.99, 2.42)
Positive on mCPR between	0.81 (−31.21, 32.83)	0.37 (−9.92, 10.66)	−0.03 (−2.84, 2.77)
**12-week Follow Up**			
Negative on mCPR between	5.36 (−68.25, 78.96)	2.58 (−10.18, 15,34)	−0.66 (−2.30, 0.98)
Positive on mCPR between	2.48 (−11.32, 16.27)	1.93 (−17.08, 20.94)	−0.50 (−2.48, 1.48)
**24-week Follow Up**			
Negative on mCPR between	−4.26 (−34.82, 26,29)	0.03 (−20.65, 20.70)	0.16 (−1.85, 2.17)
Positive on mCPR between	−8.50 (−48.75, 31.75)	−2.28 (−29.18, 24.62)	0.11 (−2.05, 2.26)

^a^A positive difference score indicates SMT group reported more pain in comparison to the AET group. A negative difference score indicates the AET group reported more pain in comparison to the SMT group. The larger the absolute value, the greater the difference.

^b^t A positive difference score indicates SMT group reported more disability in comparison to the AET group. A negative difference score indicates the AET group reported more disability in comparison to the SMT group. The larger the absolute value, the greater the difference.

^c^A positive difference score indicates SMT group reported less pain in comparison to the AET group. A negative difference score indicates the AET group reported less pain in comparison to the SMT group. The larger the absolute value, the greater the difference.

### Patient satisfaction

We found a significant mCPR x treatment arm interaction (p = .02, partial eta^2^ = .03) on the patients’ satisfaction scores. As seen in Table 6, the positive on the mCPR group was equally satisfied with the AET and SMT. In contrast, the negative on the mCPR group was more satisfied with the AET than the SMT.

**Table 6:** **Treatment satisfaction as a function of mCPR and treatment group**

**mCPR status**	**Treatment group**	
	**SMT**	**AET**
	**Mean** ^**a**^ **(±95%** **CI)**	**Mean** **(±95%** **CI)**
**Negative on mCPR**	3.65^a^ (3.42, 3.89) st dev = .76	4.14 (3.88, 4.04) st dev = .55
	n = 26	n = 21
**Positive on mCPR**	3.97 (3.80, 4.13) st dev = .58	3.96 (3.80, 4.13) st dev = .54
	n = 52	n = 49

^a^responses measured on five point Likert Scales anchored by 1 and 5. The higher the number the more satisfaction.

### Harms

Adverse event (AE) and serious adverse event (SAE) data were tracked for each of the treatment groups, AET and SMT. Adverse event data was collected at each treatment visit and patients also received phone calls during the 5, 12, and 24-week post baseline follow up period. For purpose of this protocol, an AE was defined as any undesirable medical event with new onset or significant exacerbation during the course of the study, regardless of whether or not it was considered to be related to study treatment. Each clinician rated each AE as to severity (a clinical judgment): mild, moderate or severe. An SAE was defined as any AE occurring during the study or within 30 days of conclusion of study participation resulting in any one of the following outcomes: death, life threatening persistent or significant disability/incapacity, hospitalization (when the result of an AE occurring during the study; note, hospitalization for an elective procedure or for treatment of a pre-existing condition not worsened during the study was not considered an SAE; admission to the ER for 23 hours or less was not considered a hospitalization), congenital anomaly, important medical event (i.e. an event that in the opinion of the investigator may jeopardize the participant and may require medical or surgical intervention to prevent one of the outcomes listed above). The Data Safety and Management Board (DSMB) met four times during the study (at 25%, 50%, 75%, and final enrollment), the DSMB evaluated the reported AEs and SAEs and found no issues with the reporting of these events and no trends that would require alteration of the study methods. A total of 243 AEs were reported over the course of the study with 54.7% in the AET group and 45.3% in the SMT group. Of the 133 AEs reported in the AET group, the DSMB judged 16 as definitely or probably associated with the intervention. Of the 110 AEs reported in the SMT group, the DSMB judged 14 as definitely or probably associated with the intervention. The majority of AEs that were reported consisted of musculoskeletal soreness and resolved within the study period. During the study period there were 10 SAEs reported after the start of the treatments (5 in the AET group and 5 in the SMT group), the DSMB judged that none of the SAEs were associated with the study intervention.

## Discussion

### Interpretation

Recent literature has highlighted the lack of definitive data to emerge from RCTs evaluating CLBP, with no treatment producing consistently superior outcomes [29-32]. In keeping with this previous literature and supporting our first hypothesis, we found clinically and statistically significant improvements in outcomes from baseline to follow up in the groups receiving SMT and AET, which are both recognized as evidence based interventions for CLBP [10,31].

After an initial promising start in developing treatment based classification in lower back pain, two recent reviews did not identify any studies validating the use of a treatment based classification in CLBP [33,34]. The lack of data on specific patient factors that would moderate the treatment of CLBP is what makes the current study important. Our second hypothesis was that the status on the mCPR would moderate the effectiveness of SMT. If the mCPR moderated the effectiveness of SMT, then we would have expected a significant treatment × mCPR × time interaction. We did not find significant treatment × mCPR × time interaction and so we cannot support our second hypothesis.

Our study results can be compared to the findings of Hancock et al. [35], that found the CPR performed no better than chance in identifying responsiveness to SMT among patients with acute lower back pain. Together the two studies (Hancock and our study) that attempted to apply the CPR to specific populations (Hancock et al. in acute lower back pain and ours in chronic lower back pain) suggest that the CPR, as it is able to be applied, does not seem to moderate the responsiveness to SMT of lower back pain patients, and thus suggesting a limited use of mCPR in clinical judgments of treatment selection for CLBP patients. We are aware of the fallacy of “proving” the null hypothesis of no effect, and so call for additional research to determine the effectiveness of the CPR as a clinical tool in predicting treatment responsiveness in lower back pain patients. Of particular note is the need for additional research to examine the role of other psychosocial factors in the prognosis of CLBP patients [36-39].

### Limitations

Our study is limited by factors that may need to be addressed in future trials. The first limitation is that we modified a rule that was developed to be utilized in general population of LBP patients. The original rule defined a patient as positive on the rule, if the patient scored positive on four or more of the five criteria, one of which was pain <16 days (acute pain). Since all the patients had CLBP they could not meet the mCPR criteria of pain <16 days (i.e., acute pain). Using the original rule would have been overly restrictive by drastically limiting the universe of positive mCPR patients, who would have had to score positive on all the remaining four rule criteria.

We recognize that our study is limited to two different broadly defined interventions and that this limits the extent to which one can make definitive statements about the individual treatment nuances of each. We purposefully allowed for a more broadly defined SMT treatment, in order to capture more realistically the treatments of clinicians performing manual therapy. Allowing for a more encompassing definition has been seen in other studies, both of these studies allowed the SMT groups to utilize treatments outside of high velocity low amplitude SMT [40,41]. We however, realize that this comes at a price of clouding the impact of specific types of SMT, thus limiting the direct correlation to the original CPR. Further studies may be used to address which specific aspects of the SMT (manipulative thrust or distraction) were the most effective. Another limitation is that the SMT group did allow for the recommendation of a simple stretching exercise (Cat/camel stretch); although allowing this did increase the generalizability of the study as a whole, it limits that ability to evaluate SMT alone. We also recognize the limitations due to the subjective nature of some of the assessment tools utilized in this study including the use of “deep palpation of erector spinae” and the use of “hypomobility of one or greater segments.” These assessment tools have not demonstrated reliability or validity; however they are commonly utilized measures in clinical practice. We acknowledge that the use of these criteria could introduce certain selection bias in the inclusion criteria and the designation of status on the prediction rule; however the study utilized previously reported criteria [12]. To attempt to maintain reliability however, we did use a single screening clinician to perform all baseline screening examinations. Future study should work to identify reliable and valid criteria for identification of pain and hypomobility.

### Generalizability

The current trial utilized easily administered tools (spinal mobility, hip motion, symptom characteristics and a simple questionnaire) to attempt to characterize those patients who would respond to one type of treatment over another. In addition, the interventions were designed to be easily generalizable to the typical practice of a manual therapist (PT, DC or Osteopath). This study was designed to apply a modification of a previously defined CPR and see if it was applicable to a different population and a more generalizable treatment method. This study should not be construed to discount the original CPR.

## Conclusion

While patients benefited from both SMT and AET, the mCPR did not moderate the effectiveness of SMT, as we hypothesized. Future studies are needed to better understand the specific and non-specific nature of interventions for CLBP [42-45] and also to aid the general practitioner in his/her decision on what intervention may be most appropriate. Further studies are warranted to evaluate the underlying physiological and psychological mechanisms in CLBP in order to better address these underlying abnormalities with the most effective treatment. The results of these studies may help to inform development of a new CPR that would be applicable to CLBP. There is also a need for further studies to evaluate the role of predictive factors for responsiveness for conservative interventions that will be sensitive to the role of non-specific effects of both SMT and AET.

## Abbreviations

AE: Adverse Event
AET: Active Exercise Therapy
CLBP: Chronic Lower Back Pain
CPR: Clinical Prediction Rule
CONSORT: Consolidated Standards of Reporting Trials
DC: Doctor of Chiropractic
DSMB: Data and Safety Monitoring Board
FABQ: Fear Avoidance Belief Questionnaire
HRSA: Health Resources and Services Administration
IRB: Institutional Review Board
LBP: Lower Back Pain
MCMC: Markov chain Monte Carlo
mCPR: Modified Clinical Prediction Rule
NYCC: New York Chiropractic College
ODI: Oswestry Disability Index
PT: Physical Therapist
RCT: Randomized Controlled Trial
SAE: Serious Adverse Event
SF-36: Short Form-36 Item Health Survey
SMT: Spinal Manipulative Therapy
SPSS: Statistical Package for the Social Sciences
VA: Veteran’s Affairs
VAS: Visual Analogue Scale
